# Feasibility and proof-of-concept of a combined resilience and social connection intervention for adolescents and young adults with cancer: a pilot randomized trial protocol

**DOI:** 10.1186/s40814-026-01775-0

**Published:** 2026-01-29

**Authors:** Kaitlyn M. Fladeboe, Grace Fredman, Scott H. Maurer, Chuan Zhou, Miranda C. Bradford, Joyce P. Yi-Frazier, John M. Salsman, K. Scott Baker, Molly C. Mack, Mallory R. Taylor, Abby R. Rosenberg

**Affiliations:** 1 Ben Towne Center for Childhood Cancer and Blood Disorders Research, Seattle Children’s Research Institute, Seattle, WA USA; 2 Department of Pediatrics, University of Washington School of Medicine, Seattle, WA USA; 3 Department of Pediatrics, University of Pittsburgh, Pittsburgh, PA USA; 4 Division of Palliative Medicine and Supportive Care, UPMC Children’s Hospital of Pittsburgh, Pittsburgh, PA USA; 5 Center for Child Health, Behavior, and Development, Seattle Children’s Research Institute, Seattle, WA USA; 6 Biostatistics, Epidemiology, and Analytics in Research Core, Seattle Children’s Research Institute, Seattle, WA USA; 7 Department of Supportive Oncology, Dana-Farber Cancer Institute, Boston, MA USA; 8 Department of Social Sciences and Health Policy, Wake Forest School of Medicine, Winston-Salem, NC USA; 9 Atrium Health Wake Forest Baptist Comprehensive Cancer Center, Winston Salem, NC USA; 10 Clinical Research Division, Fred Hutchinson Cancer Center, Seattle, WA USA; 11 Department of Pediatrics, Boston Children’s Hospital, Boston, MA USA; 12 Department of Pediatrics, Harvard Medical School, Boston, MA USA

**Keywords:** Intervention, Social wellbeing, Resilience, Oncology, Adolescent, Young adult

## Abstract

**Background:**

Adolescents and young adults (AYAs) with cancer experience deficits in social connection that persist into survivorship; currently, few interventions target this unmet need. The current article describes the protocol for a pilot, parallel-group randomized controlled trial of a psychosocial intervention [Promoting Resilience in Stress Management (PRISM)] that includes a new skill-based module targeting AYA social needs (SN). The aims are to (1) establish the feasibility and acceptability of the PRISM-SN–adapted program; and (2) demonstrate proof-of-concept via clinically meaningful improvements in patient-reported outcomes (PROs).

**Methods:**

We anticipate 70 AYAs will enroll and complete data collection at two sites: Seattle Children’s Hospital and UPMC Children’s Hospital of Pittsburgh. Eligible AYAs are ages 12–25 years old; diagnosed with a new malignancy < 6 months; treatment plan includes chemotherapy and/or radiation; and are English-speaking. Enrolled AYAs are randomized 1:1 to receive PRISM-SN or usual care and complete surveys at baseline and 12-week follow-up. PRISM-SN includes 5 sessions (4 standard PRISM modules + new SN module) teaching behavioral skills associated with psychosocial wellbeing. Sessions are delivered 1:1 by a trained coach, in person or virtually, 1–2 weeks apart. Feasibility will be defined based on uptake, retention, and patient-reported intervention acceptability. Proof-of-concept will be defined based on clinically meaningful change and detectable differences in PROs at 12 weeks, including social relationship coping efficacy (primary PRO of interest), social support, quality of life, resilience, anxiety, depression, and hope. Descriptive statistics and covariate-adjusted regression models will be used to assess feasibility outcomes and examine trends and between-group differences in PROs across study arms.

**Discussion:**

This pilot trial will determine feasibility of PRISM-SN in the context of a multi-site trial; provide proof-of-concept via effects of PRISM-SN on social connection outcomes; and represent an important step toward addressing an unmet need in AYA cancer care. Future directions include testing efficacy and effectiveness via larger multicenter trials.

**Trial registration:**

ClinicalTrials.gov Identifier NCT06242964

**Supplementary Information:**

The online version contains supplementary material available at 10.1186/s40814-026-01775-0.

## Background

Adolescents and young adults (AYAs) with cancer experience deficits in social connection which persist into survivorship [1–3]. Social connection is an umbrella term that encompasses the structure, function, and quality of one’s social relationships [4]. Compared to healthy peers, AYAs with cancer report lower social quality of life and social functioning at diagnosis and 2 years later [3, 5]. Many experience social isolation and insufficient social support [6–8], describe feeling distant from peers [9, 10], report challenges navigating illness-related stressors in close relationships [10, 11] and rank supportive care resources to enhance social connection as a top priority [1, 12, 13]. Given well-established links between social connection and both physical and mental health [14–17], addressing this need may be an important avenue for improving long-term outcomes.

Existing psychosocial and behavioral interventions have been limited in their ability to improve social connection among AYAs [18]. Intervention approaches have primarily included support groups [19], group-based social activities [20, 21], or group-based delivery of psychological treatments or interventions [22, 23]. Most programs target social support via connecting AYAs with one another; fewer consider support from other interpersonal relationships or target other aspects of social connection, which may limit overall success. For example, though support from other AYAs is valuable [11, 12, 24], AYAs also derive support from other consequential relationships including family members and healthy peers [10, 11, 25]. Cancer-related challenges in these relationships are common [1, 11, 25], may exceed AYAs’ still-developing socioemotional skills [26, 27], and thus contribute to a range of social connection deficits such as social isolation, poor social functioning, and inadequate support. Novel, more comprehensive approaches for addressing social connection in the AYA population are needed.

Individually delivered, skill-based interventions such as the Promoting Resilience in Stress Management (PRISM) program [28] have successfully improved psychosocial outcomes among AYAs with serious illness. Informed by stress and coping and resilience theories [29–32], Cognitive-Behavioral Therapy, and positive psychology interventions [33–39], PRISM is a lay coach-led intervention delivered in a brief, 1:1 format, teaching actionable behavioral skills associated with wellbeing [28, 40]. PRISM has been shown to be feasible, acceptable [28, 41, 42] and efficacious in improving resilience, hope, and quality-of-life and reducing distress among AYAs with cancer or other serious illness [43–46]. As designed, PRISM does not directly target social connection; however, its multi-component, skill-based framework and established success allow for adaptation and expansion. Given robust associations between social connection and other aspects of wellbeing [6, 14, 47, 48], expanding the PRISM program to include a component addressing AYAs’ social needs has the potential to increase the comprehensiveness and efficacy of the program while addressing a critical need in AYA cancer care.

We followed the ORBIT model for behavioral treatment development [49] and patient-centered intervention design principles [50] to create a novel PRISM module to enhance social connection among AYAs with cancer [51]. ORBIT is a flexible, progressive model for translating basic social sciences research to health-related behavioral treatments, with phases ranging from initial design to effectiveness testing [49]. Fulfilling the goals of ORBIT Phase I (Design: Define & Refine), we applied theories of self-efficacy and behavior change [52, 53], findings from preliminary and extant research on AYA social connection [1, 9–11, 25], and AYA stakeholder input to develop a skill-based PRISM module to enhance AYAs’ social relationship coping efficacy (SRCE) [54]. SRCE is defined as the confidence in one’s ability to engage in behaviors that foster, maintain, or enhance close social relationships in the context of cancer. SRCE represents a proximal outcome that is associated with a variety of downstream indicators of social connection (i.e., social support, social quality of life) and psychological wellbeing for individuals with cancer [54] and can be targeted through an individual behavioral intervention such as PRISM. Module content was acceptable among AYA stakeholders when delivered individually and as part of the PRISM program [51].

## Objectives and study design

This article describes the protocol of a pilot randomized-controlled trial (RCT) of the Promoting Resilience in Stress Management–Social Needs (PRISM-SN) adapted intervention among AYAs newly diagnosed with cancer**.** Our objectives are to assess the following: (1) feasibility, quantified via uptake, retention, and intervention acceptability; and (2) proof-of-concept, measured via minimal important difference on patient-reported outcomes (PROs) including SRCE (primary PRO of interest), social support, quality of life, hope, resilience, anxiety, and depression. For this pilot, parallel-group randomized trial, participants are randomized 1:1 to psychosocial usual care (UC, “control”) or PRISM-SN + UC (“intervention”) and complete PRO surveys at baseline and 12-week follow-up. We selected a pilot trial design with UC as our comparator to establish feasibility of delivering the PRISM-SN program in the context of a randomized trial and determine whether there is a signal of change in psychosocial outcomes in response to PRISM-SN compared to current standards of care. This protocol is reported following SPIRIT guidelines [55] adapted based on the CONSORT extension to pilot and feasibility trials [56] (Additional file 1).

## Method

### Participants and procedures

Participants are recruited from two academic pediatric oncology centers in the United States: Seattle Children’s Hospital (SCH) and University of Pittsburgh Medical Center (UPMC) Children’s Hospital of Pittsburgh. To be eligible, AYAs must be 12–25 years old; diagnosed with a new malignancy treated with chemotherapy and/or radiation in the past 6 months; able to speak and read English; and cognitively able to complete the intervention and surveys. Concomitant psychological or psychiatric care and/or other psychosocial interventions are permitted during the trial, though AYAs may not have had previous exposure to or be co-enrolled in other studies of the PRISM program. Potentially eligible AYAs are identified by screening outpatient clinic and inpatient rosters, and eligibility is confirmed via electronic health record review. Eligible AYAs (and a parent or legal guardian, if < 18 years old) are then approached by study staff in person during a clinic visit or inpatient stay to introduce the study and obtain informed consent (AYAs ≥ 18 years old, parents of those younger) and written assent (AYAs 12–17 years old). Strategies to promote enrollment include utilizing in-person approaches; communicating with providers regarding ideal times for approach; and providing the option to complete consent conferences virtually/using electronic signatures after the initial in-person approach.

**Fig. 1:**
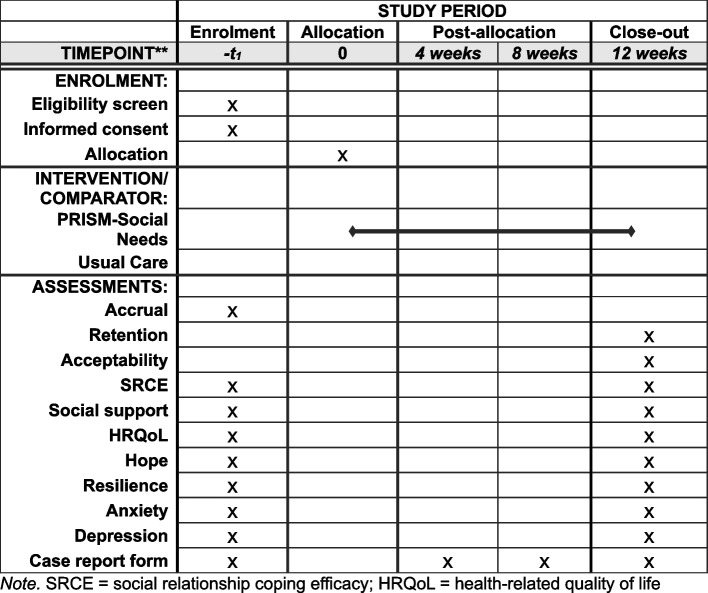
Schedule of enrollment, intervention, and assessments (SPIRIT Figure)

Participant timeline is described in Fig. 1. Enrolled participants on both arms complete a survey containing a demographic questionnaire and a PRO battery at baseline (pre-randomization) and at 12-week follow-up. Surveys are administered via Research Electronic Data Capture (REDCap) [57] or paper and pencil upon request and must be completed within 4 weeks. Incentives of $25 and $50 are provided for baseline and follow-up surveys, respectively, and participants on both arms receive a small non-monetary incentive (e.g., tote bag or water bottle with the study logo) during their participation. To promote retention and follow-up, study staff contact participants via email, phone, or in-person check-ins to notify them of an upcoming survey due date and confirm how they would like to complete the survey (e.g., email link, study tablet provided during clinic visit, paper and pencil). Thereafter, staff provide weekly reminders for outstanding surveys. Clinical information is abstracted from electronic health records each month throughout the study period, including diagnosis, diagnosis date, treatment modalities and intensity, any pre-existing or current psychiatric diagnoses, and any formal psychological treatment received during study participation. Participants may be taken off study if they request to withdraw, do not complete study activities within the specified timeframe, are unable to be reached by study staff for > 4 weeks, or are deceased before completing study activities. For participants who request to withdraw from the study, study staff ask for and document reasons for withdrawal.

### Randomization

Following baseline survey completion, AYAs are randomized 1:1 to the intervention or the control arm, with strata defined by age group (12–17 vs. 18–25 years) and study site. The randomization was constructed by the study statistician using a permuted blocks scheme with randomly varying block sizes. Study staff administer randomization assignments using REDCap [57] and maintain a randomization log; staff do not have access to the random allocation sequence. Blinding of study staff or participants is not feasible or practical given the nature of the intervention; however, study statisticians will be blinded to group assignment for data analysis.

### Intervention

#### Usual care

Participants randomized to both arms receive psychosocial usual care. At both sites, this includes an assigned social worker who conducts a comprehensive assessment at diagnosis and provides additional ad hoc services throughout treatment, with professional psychology services available upon family request or care team referral.

#### PRISM-SN intervention

Participants randomized to the intervention arm receive the PRISM-SN program in addition to UC. PRISM-SN includes an introductory meeting, five core modules (four standard PRISM modules + new social needs [SN] module), and a final family meeting. Each module is delivered as a single 30–60-min session; 1:1, in person or via videoconference; approximately 1–2 weeks apart.

PRISM-SN is delivered by trained “coaches” with a bachelor’s degree or higher who complete a standardized intervention training (i.e., 8 h of supervised didactics, role-playing scenarios, recognizing signs of psychological distress or risks of harm). Coach supervision and fidelity monitoring are conducted by the PI. Fidelity is assessed for the first 5 sessions delivered by each coach and 1 in 5 randomly selected sessions thereafter using a standardized scoring system developed for the PRISM program [28]. Coach supervision includes weekly study team check-ins; monthly group supervision meetings to provide feedback and discuss emergent concerns; and re-training as needed to address fidelity concerns.

Intervention content is described in Table 1. The introductory meeting includes discussion of the concept of resilience and review of participants’ “toolkit”, or existing skills for managing stress. Next, through each module, AYAs learn “resilience resources” or skills associated with wellbeing. Skills are taught using AYA-specific example scenarios as behavioral models and practiced in-vivo using a participant-generated example. As described elsewhere [51], the social needs module (i.e., Module 5: Connecting with Others) includes two new skills focused on identifying support and navigating relationship challenges. In the final session, participants review what was learned through the program, how to integrate new skills into their “toolkit”, and have the option to invite a parent or support person to join. To practice skills between sessions, participants receive paper “cheat-sheets” corresponding with each session and are invited to download the PRISM mobile app. App content mirrors paper cheat-sheets for all sessions except “Connecting with Others”, for which mobile app content is not yet available.

**Table 1:** The Promoting Resilience in Stress Management–Social Needs (PRISM-SN) Intervention

Session	Skill(s) and content	Delivery
Introduction + PRISM 1: Managing Stress^+^	Introducing resilience “toolkit”; Deep-breathing, mindfulness strategies	1:1
PRISM 2: Catching Negative Thoughts^+^	Recognizing and reframing negative self-talk	
PRISM 3: Setting Goals^+^	Setting SMART goals	
PRISM 4: Meaning Making^+^	Identify meaning, purpose from cancer experience	
PRISM 5: Connecting with Others^*^	Identifying support, navigating relational challenges	
Family meeting	Reviewing what was learned, discussing how loved ones can help	1:1 or group

*Note*: ^+^Standard PRISM module

^*^New social needs (SN) module

Various strategies are employed to promote uptake, adherence, and retention. Sessions may be delivered in-person or remotely based on participant preference. In-person sessions are scheduled in tandem with oncology clinic visits or inpatient stays and do not require additional hospital visits. All sessions are scheduled in advance, and participants receive reminder calls or emails one day prior. For participants with scheduling challenges or limited availability, two modules may be delivered within a single session. Intervention arm participants who request to discontinue participation are given the option to stop intervention sessions and remain in the study (and thus remain eligible to complete the follow-up survey) or withdraw from the research entirely. Any reasons provided for discontinuing sessions are documented.

### Outcomes

#### Feasibility and acceptability outcomes

Our first aim is to establish feasibility and acceptability of the PRISM-SN program. Our *a priori* feasibility outcomes include: (a) *uptake*, calculated as the proportion of AYAs enrolled out of total AYAs approached and (b) *retention*, calculated as the proportion of AYAs who remain in the study at 12 weeks out of total AYAs enrolled. PRISM-SN will be considered feasible if uptake is ≥ 70% and retention is ≥ 70%. We will also examine additional feasibility outcomes relevant to intervention delivery and adherence, including the number of sessions completed, session format and frequency, intervention length, and fidelity. *Acceptability *will be determined via the patient-reported Acceptability of Intervention Measure [58] at 12 weeks. This validated instrument includes 4 items such as “[program] meets my approval,” scored 1–5, with higher average scores reflecting more acceptability. PRISM-SN will be acceptable if ≥ 80% of AYAs on the intervention arm have an average score of ≥ 4 on this measure.

#### Patient-reported psychosocial outcomes

Our second aim is to explore trends and detectable between-group differences in psychosocial PROs at 12 weeks. Our primary PRO of interest is *Social Relationship Coping Efficacy (SRCE),* measured using the validated Cancer Behavior Inventory–Social Relationship Coping Efficacy subscale [54]. Ten items, on 1–9 Likert scale, assess confidence to engage in behaviors that maintain or enhance relationships in the context of illness. We also assess five secondary PROs. *Perceived Social Support* is measured using the Multidimensional Scale of Perceived Social Support [59]. This 12-item tool measures support from family, friends, and a significant other. *Quality of Life* is measured via the Patient-Reported Outcomes Measurement Information System (PROMIS) Pediatric Profile-25 (v2.0) [60]. This profile includes 25 items assessing health status across 7 domains: physical function mobility, anxiety, depressive symptoms, fatigue, peer relationships, pain interference, and pain intensity [61–64]; we also included an additional 4-items assessing family relationships (PROMIS Pediatric Family Relationships short-form v1.0) [65]. *Anxiety and depression* are measured using the Hospital Anxiety and Depression Scale [66]. *Self-perceived resilience* is measured using the 10-item Connor-Davidson Resilience Scale [67]. *Hope,* or the overall perception that one’s goals can be met, is measured using the 8-item Snyder Hope Scale [68].

### Data analysis and sample size

Conservatively assuming 30% attrition due to illness, death, or dropout, we anticipate recruiting 100 AYAs over 2 years, with an evaluable sample of 70 (*N* = 35 per arm). This sample size will provide a margin of error ± 11% at 95% confidence for an estimated rate of 70%; for a rate of 80%, the margin of error is ± 9% at 95% confidence. This pilot trial is not powered to detect efficacy; data will be used to demonstrate intervention feasibility and proof-of-concept and inform the design of future larger randomized trials including benchmarks for accrual, retention, and intervention adherence.

Primary statistical analyses will be conducted on an intention-to-treat basis to avoid confounding by non-random participant attrition. To determine feasibility and acceptability (Study Objective 1), benchmark proportions and descriptive statistics will be used to examine uptake and retention rates and AIM scores. To determine proof-of-concept (Study Objective 2), demographics, clinical characteristics, and PRO items will be summarized at each time point using descriptive statistics: frequencies and proportions for categorical variables, means and standard deviations for continuous variables, or the median and interquartile range if the distribution is markedly skewed. Categorical outcomes will be compared between arms using the chi-squared test, and continuous outcomes will be compared using two-sample *t*-tests. In addition, covariate-adjusted linear regression models will be used to compare PROs between study arms, in which study arm is the predictor of interest and the stratification factors, site and age, are the covariates. For PROs without an established minimal clinically important difference (MCID), we will define minimal important differences as ½ standard deviation based on comparison samples [69].

### Data management, safety, and monitoring

Study coordination and data management are based at Seattle Children’s Research Institute. Recruitment, survey, and intervention completion data for this study are collected and stored using REDCap [57], a Health Insurance Portability and Accountability Act (HIPAA) compliant platform managed by Seattle Children’s. Participant data are labeled using study identification numbers. Only study personnel with documented human subjects training and permission granted by the PI can access the database. Intervention sessions recorded for fidelity monitoring purposes are labeled with study identification numbers only and stored on password-protected secure servers at each site.

Per National Institutes of Health guidelines, a Data Safety Monitoring Board is not required given the early phase and minimal risk nature of this trial. The PI is responsible for all data and safety monitoring with support of the site PI and lead research coordinator. To ensure data quality, the PI conducts a weekly review of study databases, and staff review all surveys within 72 h for completion. The PI meets with the site PI and study staff at both sites weekly to review and discuss recruitment, data collection, and intervention delivery, and convenes quarterly meetings with an advisory committee, including the PRISM intervention developers (ARR, JPY) and experienced behavioral intervention scientists. Any protocol deviations, instances of reportable new information (RNIs), or adverse events are recorded on standard forms and reported to the IRB of record for review. If any serious adverse events occur, these will be reported in writing to the IRB of record and the study sponsor and will trigger an immediate suspension of the research.

The PRISM-SN intervention addresses potentially sensitive subject matter. To ensure participant safety, PRISM coaches are trained to recognize signs of distress, concerning language, and potential for harm to self or others that may arise during sessions. If these instances occur, coaches are instructed to immediately notify the PI and the participant’s care team. Participants are informed that confidentiality may be breached in these instances. Given the early-stage nature of this trial, no interim analyses were planned.

### Research ethics and dissemination

A reliance agreement was established for the participating site to rely on the primary site’s IRB (Seattle Children’s Hospital); all study procedures were approved by the Seattle Children’s Hospital IRB (IRB00000277; IRB00009311). Any protocol amendments approved by the SCH IRB are communicated to the participating site via email; a shared study folder accessible by both sites is maintained by the lead research coordinator and contains all up-to-date study documents.

Study findings will be disseminated via publications and presentations at national and/or international meetings. Authors will include study investigators, collaborators, and staff; PRISM intervention developers; and members of the study advisory committee. The final study dataset will be de-identified and accessible to the PI and to trained study staff with IRB approval. Given the sensitive nature of the study population, participant-level data will not be made public to ensure privacy; any requests to use de-identified study data for secondary analyses must be submitted in writing to the PI and to the IRB for approval. The full study protocol and statistical code used in primary analyses may be published in public repositories per journal requirements.

## Discussion

This paper describes the protocol for a pilot randomized trial to demonstrate feasibility and proof-of-concept for the Promoting Resilience in Stress Management–Social Needs (PRISM-SN) intervention. PRISM-SN is an adaptation of an established psychosocial program [28, 43] including a new component designed to enhance social connection for AYAs newly diagnosed with cancer [51]. Anticipated outcomes from this study include feasibility, measured by uptake, retention, and intervention acceptability, and proof-of-concept, determined based on improvements in patient-reported social outcomes at 12-week follow-up.

This study has important strengths related to the intervention and study design. Leveraging an already established and successful program [43, 45, 46, 70], PRISM-SN applies a novel, developmentally informed, skills-based approach to target social connection, a previously unmet need for AYAs with cancer. In addition, the two-site pilot randomized trial design has the potential to provide stronger evidence of feasibility compared to smaller single-arm studies. This study will demonstrate the feasibility of delivering the PRISM-SN program; of recruiting and retaining AYAs in both the UC and intervention trial arms; and of implementing this protocol at multiple national sites. In addition, feasibility outcomes from this study will inform benchmarks for uptake, retention, intervention adherence, and data completion for a subsequent multi-site efficacy trial.

Limitations of this protocol should also be noted. First, generalizability may be limited by study sites and the patient populations they serve. Both sites are academic, pediatric-based AYA oncology centers who serve patient populations with limited racial and ethnic diversity. Subsequent trials of this program will need to recruit larger, more diverse samples and determine whether this protocol is feasible at community-based or adult AYA cancer centers. Second, PRISM-SN is only available in English; translation to other languages is an important future direction. Third, this pilot trial will not be powered to test whether delivery adaptations to promote accessibility (i.e., in-person vs. virtual sessions, combining modules) affect response to the program.

Future directions for this work include subsequent trials to determine whether PRISM-SN can improve social connection among AYAs in a large, diverse sample; examining psychosocial factors such as social anxiety that may influence response to the program; determining the added value of PRISM-SN compared to standard PRISM; examining implementation outcomes such as cost-effectiveness; and exploring the utility of the “Connecting with Others” skills for other populations or when combined with other supportive care programs.

Given the well-established risk for social connection deficits among AYAs with cancer and the limited availability of comprehensive interventions, novel programs including social health targets are needed. PRISM-SN has the potential to address this gap, enhance the comprehensiveness of the PRISM program, and improve overall psychosocial adaptation within the AYA oncology population.

## Supplementary Information

Additional file 1.

## Data Availability

The datasets generated by this study will not be publicly available due to due to sensitive nature of the participant population and potential for individual privacy to be compromised but are available from the corresponding author on reasonable request.

## Abbreviations

AYA: Adolescent and young adult
PRISM: Promoting Resilience in Stress Management
PRISM-SN: Promoting Resilience in Stress Management–Social Needs
ORBIT: Obesity-Related Behavioral Intervention Trials
SRCE: Social Relationship Coping Efficacy
PRO: Patient-Reported Outcome
UC: Usual Care
REDCap: Research Electronic Data Capture
PROMIS: Patient-Reported Outcomes Measurement Information System
MCID: Minimal Clinically Important Difference
PHI: Protected Health Information
IRB: Institutional Review Board
